# A phase-field study to explore the nature of the morphological instability of Kirkendall voids in complex alloys

**DOI:** 10.1038/s41598-024-81532-6

**Published:** 2024-12-16

**Authors:** Ahmadreza Riyahi khorasgani, Ingo Steinbach, Bettina Camin, Julia Kundin

**Affiliations:** 1 Interdisciplinary Centre for Advanced Materials Simulation (ICAMS), Ruhr-Universität Bochum, Universitätsstr. 150, 44801 Bochum, NRW Germany; 2 Hochschule Bremerhaven, 27568 Bremerhaven, Germany

**Keywords:** Morphological instability, Kirkendall voids, Mullins–Sekerka linear stability, Phase field, Diffusion, Engineering, Materials science

## Abstract

The present research explores theoretical and computational aspects of the morphological instability of Kirkendall voids induced by a directed flux of vacancies. A quantitative phase-field model is coupled with a multi-component diffusion model and CALPHAD-type thermodynamic and kinetic databases to obtain a meso-scale description of Kirkendall void morphologies under isothermal annealing. The material under investigation is a diffusion couple consisting of a multi-phase multi-component single-crystal Ni-based superalloy on one side and pure Ni on the other side. The flux of the fastest diffuser in the superalloy, Al, towards the pure Ni causes a strong flux of vacancies in the opposite direction. This directed flux of vacancies leads to morphologically instable growth of voids. Phase-field simulations are performed in two (2D) and three dimensions (3D) to understand these instabilities, and the results are compared with experimental observations obtained by synchrotron X-ray tomography. Finally, the simulation results are analyzed with respect to the Mullins–Sekerka linear stability criterion.

## Introduction

Morphological instability of a phase occurs when the original morphology becomes instable and a new morphology is established^1^. A rigorous definition of morphological instability is presented in an early work by Sekerka^2^. He argued that for a phase transformation to take place, certain transport phenomena such as diffusion of chemical species and/or heat must occur within the corresponding phases. On the other hand, continuity and conservation laws must be fulfilled at the interface of the separated phases. Suppose that there exists an exact theoretical solution to this physical problem, where the interface is assumed to be “smooth”. If this idealized system is subjected to “perturbations”, the consequent changes in the morphology of the interface may either be enhanced or diminished, leading to morphological instability or stability, respectively.^2^.

A fundamental study related to morphological instability is rooted in the experiments conducted, in 1953, by Rutter et al.^3^. They focused on unidirectional solidification in a dilute alloy, where the solid–liquid interface deviated from the expected planar form^4^ so they proposed an interpretation aligned with the “constitutional supercooling” principle. According to this principle, rejection of impurities from the solidified front by the neighboring melt causes the equilibrium solidification temperature to exceed the actual temperature of the melt, with consequent spontaneous growth of the solid into the melt. That explanation, however, neglected some key information such as the influence of surface tension and the scale of the perturbation causing instability.

One decade after these experimental observations, Mullins and Sekerka^4^ postulated an interface stability/instability theory that took into account surface tension. According to their theory, it can be assumed that the interface moves so slowly that it remains stationary within the time required for diffusion relaxation. In the case of a pure melt and small perturbations, Mullins and Sekerka linear stability analysis recognizes thermal effects as the destabilizing factor, as more latent heat flows into the supercooled liquid than into the solid, whereas the capillary effect of the interfacial energy is found to be the stabilizing factor. Therefore, stability depends on the competition of heat flow and capillarity effects. On the other hand, in the case of alloys solidifying along a temperature gradient within the liquid, the solute has generally a destabilizing effect, competing with thermal and capillary effects as stabilizing factors^1^. Mullins and Sekerka concluded that when the corresponding calculations predict instability, there is always a sinusoidal perturbation with a wavelength below which wave perturbation vanishes and above which perturbation grows. On the contrary, if the calculations predict stability, then no perturbations can grow^5^. This linear stability analysis is expressed in terms of stability length defined by

1 $$\begin{aligned} \lambda _s = 2 \pi \alpha \sqrt{l_{\textrm{dif}} d_0}, \end{aligned}$$

where $\alpha$ is a numerical constant; $l_{\textrm{dif}}$ is the diffusion length; $d_0$ is the capillary length; and $\lambda _s$ entitled “stability length” is the minimum wave length of a sinusoidal perturbation on a planar interface above which the amplitude of the perturbation will accelerate, i.e., the interface becomes instable. For instance, in the case of a directed flux of species, the Mullins–Sekerka instability describes enhanced amplification of small perturbations at the interface during a phase transformation, driven by the flux of species. This will generate irregular patterns rather than a smooth and stable interface.

However, the original version of linear stability analysis has several shortcomings, including the assumption of isotropic surface energy. In fact, without anisotropy, linear morphological stability theory is improper in the sense that perturbations in any direction transverse to the crystal growth direction have the same stability characteristics; thus, it is necessary to impose anisotropy of either the surface tension or interface kinetics^6^. An interesting analytical work by Coriell and Sekerka^5^ demonstrated that anisotropy of interfacial kinetics could induce a sinusoidal perturbation to translate parallel to an unperturbed interface as the perturbation increases in amplitude. The peaks of such a perturbation will grow normal to the unperturbed interface; this is thought to be the main reason for preferred directions during dendrite growth.

In a critical review^7^, Langer analyzed the Mullins–Sekerka instability criterion for planar interfaces, directional solidification, growth of spherical particles, and dendritic growth, extending the models to depict side-branching and tip-splitting instabilities observed in growing dendrites. Langer argued that linear instability analysis could not satisfactorily determine the underlying causes of the instability of dendrite tips. Therefore, for evaluation of dendritic growth, he devised a dimensionless stability parameter

2 $$\begin{aligned} \sigma ^* = \dfrac{2d_0D}{v_{\textrm{tip}} \rho _{\textrm{tip}}^2}, \end{aligned}$$

where $\rho _{\textrm{tip}}$ is the tip radius, $v_{\textrm{tip}}$ is the tip velocity, and *D* is the diffusion coefficient in the melt. This provided a useful criterion to determine whether tip-splitting or a stable tip will occur during dendrite formation. This parameter can also be used to realize if dendrite growth, which is itself a morphological instable form of the initial morphology, follows a stable or instable trend. According to the “marginal instability” hypothesis introduced by Langer, there is only one value of $\sigma ^*$ for which a dendrite is completely stable, i.e., no tip splitting takes place. If $\sigma ^*$ is smaller than this critical value, then the dendrite becomes instable with respect to tip-splitting, and subsequently side branching will occur. Together with Müller-Krumbhaar, he identified the value $\sigma ^*=0.026$, as an approximated fit to experiments, for metals with cubic anisotropy.

In this regard, there has also been a numerical study by Karma and Rappel^8^ in which they demonstrate that a phase-field model could reproduce the dependency of growth velocity on the tip radius and dendrite shape. They find that the values of $\sigma ^*$ in phase-field model simulations with different anisotropy strengths show a good agreement with predictions obtained by using numerical solubility theory and experiments. In fact, in marginal stability theory, the effects of interface anisotropy are incorporated implicitly through parameters such as the capillary length and stability. These parameters allow anisotropy to indirectly affect the outcomes predicted by the theory. However, more advanced models such as those based on numerical and linear solubility theories include anisotropy explicitly by introducing anisotropic surface tension terms into the interface dynamics equations^9^.

Analogous to the instability of dendritic growth in melts, we can apply similar concepts of instability to the growth and dissolution of Kirkendall voids. As suggested in the pioneering works by Kirkendall and Smigelskas^10,11^, different elements can migrate at non-equal rates during diffusion in a compositionally graded material. Such a diffusion process will direct the flux of vacancies from the side of the lower fluxes to the side of the higher fluxes in order to balance the total fluxes. This event, so-called “Kirkendall effect”, is an indicator of vacancy-mediated mechanism for interdiffusion. The Kirkendall phenomenon can manifest itself as local volume change, displacement of the reaction interface, or even creation of pores, referred to as “Kirkendall voids”^12^. Sources and sinks for creation or destruction of lattice sites are essential requirements for the maintenance of this process. These sources/sinks for vacancies could be material defects such as dislocations, grain boundaries, or pores. A widely accepted mechanism for creation of the Kirkendall voids is annihilation of vacancies on available vacancy sinks.

Morphological instability of solid–void interfaces has long been known from a theoretical point of view, as discussed in an early work by Martin et al.^13^ that thoroughly investigated the breakthrough of voids in a CoO ceramic film subjected to a medium with different oxygen potentials on either side of the film. Martin et al. showed that the oxidation on one side and the evaporation on the other side of the film pushed a flux of Co+ from a lower to a higher oxygen potential, resulting in a flux of vacancies in the opposite direction.

Although morphological instability in solidification has been the subject of many extensive studies, the morphological instability of Kirkendall voids has not yet been questioned or explored in detail. Borrowing similar principles of solidification, we propose that “supersaturation of vacancies” in the case of Kirkendall void instabilities functions in the same way as “constitutional supercooling” in solidification. This phenomenon has been approached from a theoretical and experimental perspective^13^, but there has not been any thorough simulation effort in this context, particularly for complex systems such as alloys. The current work aims to evaluate how physical and phenomenological parameters affect Kirkendall void instability. We apply a computational framework to advance our quantitative understanding of the morphological instability of Kirkendall voids and gain deeper insights into the corresponding effects.

The main modeling methodology used in this work is based on the phase-field (PF) method, which is a computational tool used to simulate interface dynamics and identify complex interface patterns^14,15^. The PF method has been used in several studies to simulate material swelling and pore formation with vacancy supersaturation due to irradiation^16–20^. However, these modeling efforts do not consider the problem of morphological instability, specially that they are limited to simple systems and don’t cover complex alloys.

**Fig. 1:**
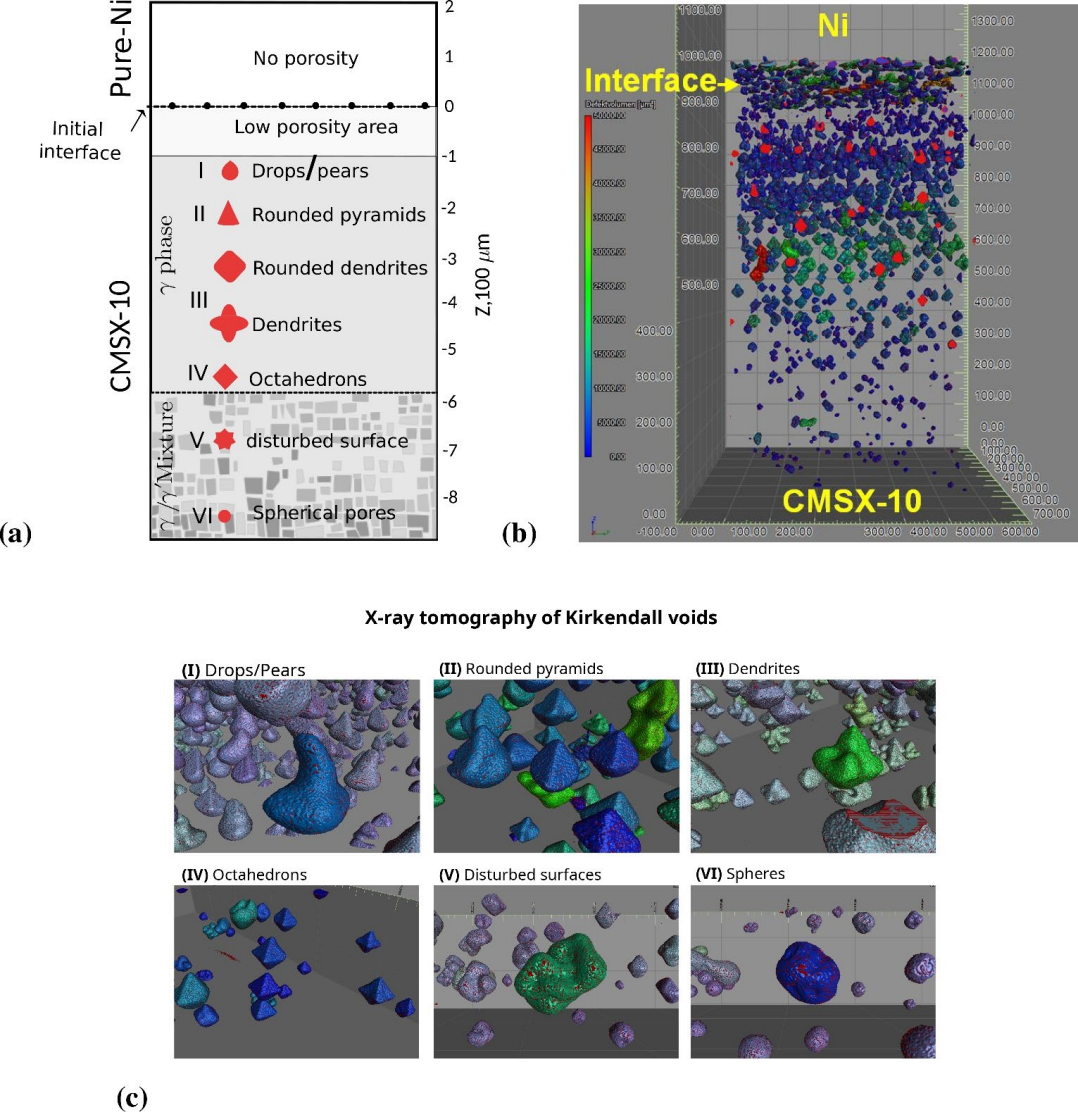
Preliminary experiment observations for CMSX-10/Ni diffusion couple under isothermal annealing at T = 1523 K for 192 h. (**a**) Schematic summary of Kirkendall voids morphologies, with regard to the 3D X-ray tomography of real voids^23^. (**b**) Synchrotron X-ray tomography of Kirkendall voids, ranging nearly from 10 to 50 $\mu$m, on CMSX-10 side (small pores at the interface are due to welding of two sides and not Kirkendall voids). (**c**) Synchrotron X-ray tomography of Kirkendall voids at different positions of the sample, as indicted in fig (**a**).

We select CMSX-10, a multi-component multi-phase single-crystal alloy, for this research, owing to its technological importance and complexity. Kirkendall void formation in CMSX-10/Ni diffusion couples after annealing at different temperatures has been extensively studied from an experimental perspective^21^. It has been demonstrated that considerable dissolution of $\gamma ^\prime$ occurs in the $\gamma$ matrix due to the rapid diffusion of Al element, as the fastest-diffusing element in this system, from the CMSX-10 side to the pure Ni side. The high fluxes of all elements are balanced by a flux of vacancies in the opposite direction of Al diffusion. This process switches on the sources of vacancies (here, edge dislocations) on the Ni side and the sinks of vacancies (here, pores) on the CMSX-10 side to generate and annihilate vacancies, respectively; for more detail, see Refs.^21,22^. Figure 1 shows a graphical summary of void morphology along a CMSX-10/Ni sample, based on preliminary X-ray tomography experiments conducted in a previous work^23^. These experimental observations illustrate a variety of interesting morphologies of pores, including dendritic, octahedral, pyramidal, cup-like, cuboidal, and spherical ones. The form and the size of pores depend on their distance from the initial interface between the alloy and pure Ni, i.e., the gradient of composition. In the current work, an equivalent alloy AlCoCrTaNi is investigated as in our previous works^22,24^. Despite the fact that precise diffusion phenomena in CMSX-10 are not definitely similar to the ones in this equivalent alloy, the interdiffusion of the main five elements follow the same trend as in real system, CMSX-10. Notably, the selection of the composition values mentioned in Table 2 predict the correct values for fluxes, specially for the dominant diffuser element (Al), as observed in the real system^22,24^. A future work is planned to simulate interdiffusion of all elements in the real system CMSX-10. Based on the phase-field scheme, in this work, we investigate the impact of the key parameters in the models. For instance, surface and kinetic anisotropy are important factors that contribute to the governing equations described here. In addition to anisotropy effects, factors including partitioning and the diffusion coefficient of vacancies influence morphological instability as well; these are all explored in the phase-field based simulations in the present research. Moreover, it is worthy mentioning that the internal stresses, caused by the Kirkendall effect, requires more complex models and is ignored in current treatment. One interesting work by Levitas^25^ shows how internal stresses can remarkably influence equilibrium concentration of vacancies as well as kinetics of nucleation and growth of Kikendall nano-voids.

In contrast with our previous work^22^, the current work covers a full parametric study of the models (Case I), 3D simulations of pores (Case II) and realistic-size diffusion couple simulation (Case III), which were all missing in our previous paper. Additionally, the phase-field simulation results are accompanied by Mullins–Sekerka analysis to shed light on the morphological instability. The findings presented here will provide material scientists with new insights into the complex morphology of Kirkendall voids.

## Methods

### Phase-field model of void evolution

In the current study, PF method plays a pivotal role in modeling morphologies of Kirkendall voids. The PF model employed here is based on our previously developed model^22^ entitled “fast model of void evolution ” (fast MVE). It utilizes the theory of crystal growth in melts with four-fold surface energy anisotropy. Furthermore, the model is coupled to accurately assessed CALPHAD databases^26–30^.

Fast MVE consists of two parts: the phase-field part and the diffusion-couple part.

***Phase-field part***

The phase-field scalar variable $\phi (x,t)$, as function of space and time indicates the presence of the growing phase. In our formulation, $\phi =1$ denotes the “pore phase” (terms “void” and “pore”, also indicated by “pore phase”, are used equivalently in this paper), whereas $\phi =0$ denotes the “bulk phase” representing a homogenized mixture of $\gamma /\gamma ^\prime$ phases within the superalloy.

The time evolution equation of the phase-field variable is constructed as

3 $$\begin{aligned} \dfrac{\partial \phi }{\partial t}&= M_{\phi }^*(\textbf{n})\left[ \sigma ({\textbf{n}}) \left( \nabla ^2\phi + \frac{\pi ^2 }{\eta ^2}(\phi -\frac{1}{2}) \right) + \Psi ({\textbf{n}}) \right. \nonumber \\&\quad \left. + \frac{\pi }{\eta }\sqrt{\phi (1- \phi )}\, X_{\textrm{b}}\Delta y_{\textrm{V}}^\textrm{eq}\dfrac{ ( y_{\textrm{V}}-y^\textrm{eq}_\textrm{V})}{\xi (\phi )}\right] , \end{aligned}$$

4 $$\begin{aligned} \Psi ({{\textbf{n}}})&= (\nabla \sigma ({{\textbf{n}}}))\cdot (\nabla \phi ) + \nabla \cdot \left( |\nabla \phi |^2 \frac{\partial \sigma ({\textbf{n}})}{\partial \nabla \phi }\right) . \end{aligned}$$

Here, $M_{\phi }^*({\textbf{n}})$ is the anisotropic interface mobility, $\sigma ({\textbf{n}})$ is the anisotropic surface energy, $\Delta y_{\textrm{V}}^\textrm{eq}=y_{\textrm{V}}^\mathrm{eq, \mathrm p} - y_{\textrm{V}}^\mathrm{eq, \mathrm b}$ is the difference between the equilibrium site fractions in the pore and bulk phases, ${\textbf{n}}$ is the normal vector to the interface, and $\xi (\phi )=(k\phi + (1-\phi ))$ is the interpolation function. The partition coefficient *k* is defined as a ratio between the thermodynamic factors in pore and bulk phases $k = \dfrac{X_{\textrm{p}}}{X_{\textrm{b}}}$. The term $\Psi ({\textbf{n}})$, which is a function of $\sigma ({\textbf{n}})$, is responsible for the cubic anisotropy of the surface energy. The derivation of this term can be found in Ref.^22^. The interface width $\eta$ is defined as a free parameter that is large enough for numerical convenience but small enough to resolve the characteristic lengths of interest^31^.

Following the thin interface limit using the relation given in Refs.^22,32^, the interface mobility is calculated as

5 $$\begin{aligned} M_{\phi } = \frac{ D_{\textrm{V}}}{a_2(\eta /\pi ) X_{\textrm{b}} (\Delta y_\textrm{V}^{eq})^2}, \end{aligned}$$

where $a_2=0.361$ is the numerical constant calculated for the double obstacle potential^33^.

The diffusion equation for vacancies in the phase-field part is

6 $$\begin{aligned} \frac{ \partial y_{\textrm{V}}}{\partial t}&= \nabla \cdot \left[ D_{\textrm{V}}(1-\phi ) \nabla \frac{( y_{\textrm{V}} -y^\textrm{eq}_{\textrm{V}})}{\xi (\phi )} + {\textbf{j}}_{\textrm{at}}\right] + r_p\dot{q}^\textrm{DC}_{\textrm{V}}(1-\phi ), \end{aligned}$$

where $\dot{q}^\textrm{DC}_{\textrm{V}}$ is the vacancy source term calculated in the diffusion-couple part of the model. In fact, this source term of vacancies is produced by the gradient of the vacancies flux. The parameter $r_p$ defines the contribution of voids to the total set of sinks on the left side of the diffusion couple. In this work, we assume $r_p=0.5$. The diffusion coefficient of vacancies $D_{\textrm{V0}}$ is assumed to be constant and equal to the average of diffusion coefficients of all elements on the left side of the diffusion couple.

***Diffusion-couple part***

The real composition profiles of elements in the diffusion couple are calculated in the diffusion-couple part by means of standard diffusion equations of components with respect to the reference element Ni, i.e.,

7 $$\begin{aligned} \frac{ \partial x_k}{\partial t}&= \frac{\partial }{\partial z}\left[ \sum _{j=1}^{n-1} {{\tilde{D}}}^\textrm{Ni}_{kj} \frac{\partial x_j}{\partial z}\right] \end{aligned}$$

whereas the mole fraction of Ni is determined as $x_{\textrm{Ni}} = 1- \sum _{j=1}^{n-1}x_k$. The equations are solved in one dimension (1D) in order to accelerate the simulations.

The interdiffusion coefficients are defined as

8 $$\begin{aligned} {{\tilde{D}}}_{kj}^\textrm{Ni}&= {{\tilde{D}}}_{kj}- {{\tilde{D}}}_{k\mathrm Ni}, \end{aligned}$$

9 $$\begin{aligned} {{\tilde{D}}}_{kj}&= \sum _{i=1}^n(\delta _{ik} -x_k)x_i M_i \Phi _{ij}, \end{aligned}$$

where $M_i$ are the atomic mobilities and $\Phi _{ij}=\dfrac{\partial \mu _{i}}{\partial x_{j}}$ are the thermodynamic factors of components. The interdiffusion coefficients, ${{\tilde{D}}}_{kj}^\textrm{Ni}$, in the diffusion-couple part are estimated at each grid point and time step.

The source term of vacancies^22^ is obtained by

10 $$\begin{aligned} \dot{q}^\textrm{DC}_{\textrm{V}} (0,0,z) = - V_{\textrm{m}}\frac{\partial J_\textrm{V}}{\partial z}, \, \text { if } \frac{\partial J_{\textrm{V}}}{\partial z} < 0, \end{aligned}$$

where $$J_{\textrm{V}} = -\sum _{k=1}^{n} J_k$$ and

11 $$\begin{aligned} J_k = -\frac{1}{V_{\textrm{m}}}\sum _{j=1}^{n} D^\textrm{I}_{kj} \frac{\partial x_j}{\partial z} . \end{aligned}$$

In the present work, the average molar volume $V_{\textrm{m}}$, for all phases, is approximated as a constant^34^. Moreover, it is assumed there would be sufficient amount of sources and sinks for vacancies such as dislocations/pores to allow the vacancy concentration to reach its equilibrium value^35,36^.

In each simulation step the 1D source term is converted to 2D and 3D by the formula

12 $$\begin{aligned} \dot{q}^\textrm{DC}_{\textrm{V}} (x,y,z) = \dot{q}^\textrm{DC}_{\textrm{V}} (0,0,z). \end{aligned}$$

The main parameters of the models used in the simulations are listed in Table 1.

**Table 1:** Model parameters for simulation for system alloy AlCoCrTaNi/Ni diffusion couple.

Parameter	Symbol	Value	Units
Grid spacing	$\Delta x$	$1\times 10^{-6}$	m
Time step	$\Delta t$	5	s
Average diffusivity of vacancies	$D_{\textrm{V0}}$	$4\times 10^{-14}$	${\textrm{m}}^2 \,{\mathrm{(s)}}^{-1}$
Average interface mobility	$M^{\phi }$	$8\times 10^{-16}$	${\textrm{m}}^4\, {\mathrm{(J s)}}^{-1}$
Interface width	$\eta$	$5 \Delta x$	m
Surface energy (pure Ni)	$\sigma _0$	2.34	J ${\textrm{m}}^{-2}$
Anisotropy strength	$\varepsilon _4$	0.023	–
Thermodynamic factor (bulk phase)	$X_{\textrm{b}}$	$6\times 10^{9}$	J/(${\textrm{m}}^3\, {\mathrm{s.f.}}^2$)
Vacancy equilibrium fraction in bulk phase	$y_{\textrm{V}}^\textrm{eq,b}$	0.01	site fraction (s.f.)
Vacancy equilibrium fraction in pore phase	$y_{\textrm{V}}^\textrm{eq,p}$	0.99	s.f.
Interface width of mixture region	$\omega$	$2\times 10^{-5}$	m
Molar volume	$V_{\textrm{m}}$	$7\times 10^{-6}$	${\textrm{m}}^3$

### Modeling of pore nucleation

The physical mechanism of pore nucleation is assumed to be a heterogeneous on the randomly distributed defects (here edge dislocations) in the alloy. We assume that sufficient number of nucleation sites with varying activity (nucleation barrier), based on the model of Quested and Greer^37^ are available in the material. According to this model, the activation is considered if the supersaturation (or source rate) of vacancies locally exceeds a nucleation barrier. However, for Case III as real-size sample, we utilized randomly distributed nucleation sites. The pore seeds begin to grow when the source term at these sites surpasses a critical threshold. This threshold is randomly assigned within the range of ${\dot{q}}^\textrm{DC}_{\textrm{V}} (z) = 1 \times 10^{-10} \text {s}^{-1}$ to $4 \times 10^{-10} \text {s}^{-1}$.

## Results

The models described in the *Methods* section are implemented by the authors in the OpenPhase software library^38^. As an equivalent to the experimental alloy system CMSX-10, we use the hypothetical AlCoCrTaNi alloy, the corresponding chemical composition of which is listed in Table 2. This alloy contains only five elements, enabling us to perform faster simulations^22^. The open-source thermodynamic databases from refs.^26,39^ and atomic mobility parameters from refs.^27–30^ are coupled to these models. Both databases are constructed using the CALPHAD technique^40^.

Simulations are categorized as Cases I, II, and III. Results for Case I show how the initial spherical nuclei of a single void inside a small box becomes instable during evolution and forms various patterns, depending on the values of the some modeling parameters such as the vacancy diffusion coefficient, surface anisotropy etc. Results for Case II depict 3D simulations for a single void, and for three voids to show the effects of their interactions, whereas results for Case III are for 2D simulations of multiple voids in a real-size diffusion couple.

**Table 2:** Initial site fraction of components in hypothetical alloy AlCoCrTaNi, equivalent to CMSX-10 alloy.

	Al	Co	Cr	Ta	Ni
Hypothetical CMSX-10	0.14	0.023	0.028	0.058	0.751

### Case I: 2D simulations of morphological instability of single Kirkendall voids

**Fig. 2:**
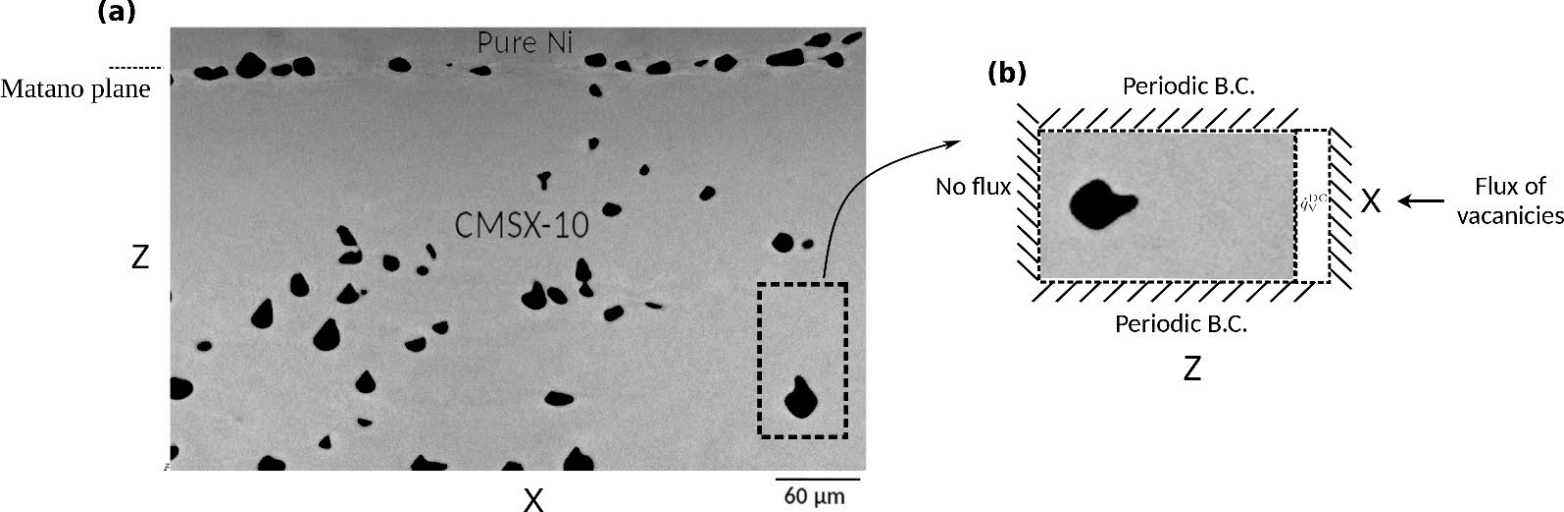
Sub-domain accommodating a single void under new boundary conditions. (**a**) Light microscope image of a diffusion couple, annealed at T=1523K for 192 h, exhibiting Kirkendall voids on CMSX-10 side (the pores along dashed line at the interface, Matano plane, are due to welding of two sides). (**b**) For case I, a sub-domain including one single pore is considered and subsequent boundary conditions due to extraction from the diffusion couple are inserted accordingly.

We begin with a study of the morphological instability of a single Kirkendall void which is inside a much smaller box compared with the real diffusion couple. These 2D tests in small boxes are conducted using the phase-field model equations with a constant value for the “vacancy source term”, denoted by $\dot{q}^\textrm{DC}_{\textrm{V}}$ in Eq. (6). Based on the simulation strategy used in Case I, we select a small sub-domain extracted from the overall domain of a diffusion couple sample, accommodating a single void, subject to subsequent new boundary conditions as illustrated in Fig. 2. In this figure, the Kirkendall pores are formed on the left side of interface of diffusion couple AlCoCrTaNi/Ni while left side, i.e., pure Ni is not shown. The Matano plane, i.e., the plane over which net flux of atoms vanishes is overlapped with welding line of two sides.

The effects of the main variables in the model equations are studied individually. The results for various scenarios are shown as 2D fields of the vacancy fraction inside pores and bulk matrix in Figs. 4, 5, 6, 7, 8 and 9. All results are achieved for 192 h of annealing at 1523 K.

Different set-ups for the 2D simulations are used, with various diffusion coefficients for vacancies and interface mobilities. The size of each box is chosen to be 60 $\upmu$m $\times$ 120 $\upmu$m. The grid spacing is equal in both directions, i.e., $\Delta x=\Delta z$, the interface width is $\eta = 5$, and $\Delta x$ as well as the time step $\Delta t$ are selected accordingly. The reference values for parameters are given as $D_\textrm{V}^0=4\times 10^{-14}\, {\textrm{m}}^2{\textrm{s}}^{-1}$, $M_\phi ^0=8\times 10^{-16}$ ${\textrm{m}}^4\, {\mathrm{(J s)}}^{-1}$ (in some literature, interface mobility is represented by $\mu$), $\Delta t_0 =5$ s, $\Delta x_0 =1$ $\upmu$m. Note that to maintain the same system size for different grid spacings, the number of grid points should change correspondingly. For example, for $\Delta x =\Delta x_0$, the box will have $60 \times 120$ grid points^2^; for $\Delta x =\Delta x_0/2$, it will have $120 \times 240$ grid points^2^.

The initial radius of the pore nucleus is selected as $2\Delta x$ and positioned at $x=z=30$ $\upmu$m if $\Delta x =\Delta x_0$, with respect to the left end of the box. Periodic boundary conditions are applied in the vertical *x*-direction, and no flux boundary conditions are employed on the left boundary of the box in the *z*-direction.

To fulfill the new boundary conditions for sub-domains, we need to reproduce the real rate of vacancy creation so that a constant vacancy “source rate” could be assumed on the right boundary in the *z* direction (with vacancies pumping from the right side, i.e., pure Ni). This value is evaluated as the integral over the source term of vacancies, calculated by Eq.  (10).

13 $$\begin{aligned} \dot{Q}_{\textrm{V}}= \int _0^{L_z} \dot{q}^\textrm{DC}_{\textrm{V}}(z) dz , \end{aligned}$$

where $L_z$ is the box size in the *z*-direction. The “accumulation rate” $\dot{Q}_{\textrm{V}}=2\times 10^{-11}\, {\textrm{ms}}^{-1}$ is estimated as a mean value through modeling of the annealing process in the diffusion couple AlCoCrTaNi/Ni. Then the constant source term, $\dot{q}^\textrm{DC}_{\textrm{V}}(\text {right boundary}) =\dot{Q}_{\textrm{V}}/\Delta z$, is applied in the diffusion Eq. (6) on the right boundary of the simulation box. It means that the source term is non-zero only on the right boundary, of the thickness $\Delta z$, and zero otherwise.

**Fig. 3:**
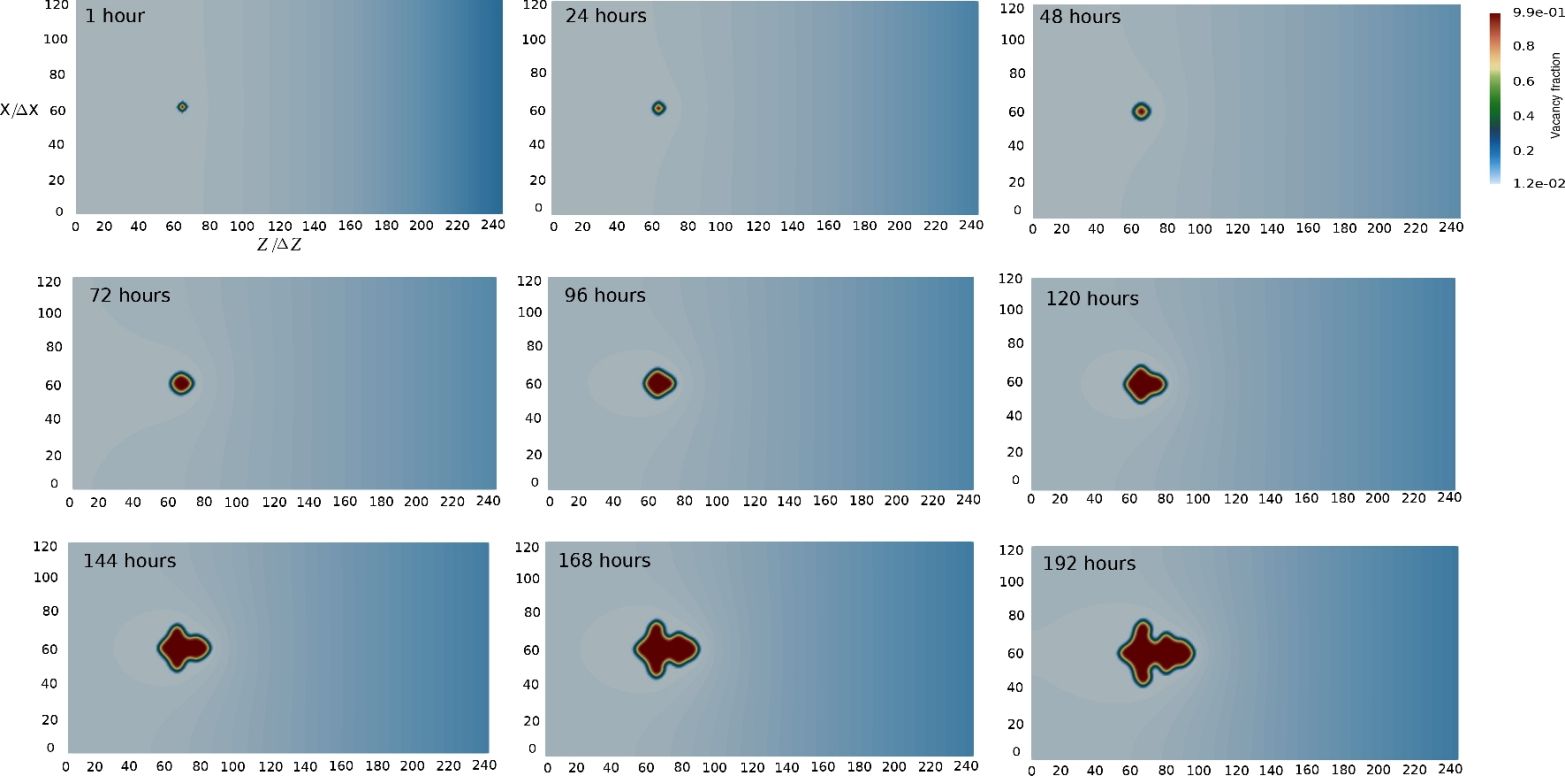
Time evolution of an exemplary dendrite void represented by successive snapshots showing different times of annealing times. $D_{\textrm{V}} =D_{\textrm{V}}^0/5$, $k=10$.

Before considering the impact of parameters on the pore morphology, we present an exemplary simulation in Fig. 3, illustrating time evolution of a dendrite, consistent with Fig. 6d. As seen in the Fig. 3, an instantaneous change of morphology begins after around 120 h. Prior to this stage, the pore maintains its circular smooth form.

(i) Impact of anisotropy

For modeling of void growth, we employ the four-fold cubic anisotropy, which is generally accepted and well-established for metals^32,33^. The effect of anisotropy is given in the phase-field Eq. (3) through two parameters of surface energy and interface mobility (kinetic anisotropy).

The anisotropic surface energy is calculated as

14 $$\begin{aligned} \sigma ({\textbf{n}}) = \sigma _0 [1+\varepsilon _4\cos (4\theta )], \end{aligned}$$

where $\varepsilon _4$ is the anisotropy strength and $\theta$ is the inclination angle^22^.

In the case of kinetic anisotropy, the anisotropic interface mobility can be calculated as a reformulation of that given in ref.^33^:

15 $$\begin{aligned} M_{\phi }^*({\textbf{n}})= M_{\phi } [1+15\varepsilon _4\cos (4\theta )], \end{aligned}$$

where $M_{\phi }$ is the isotropic interface mobility.

**Fig. 4:**
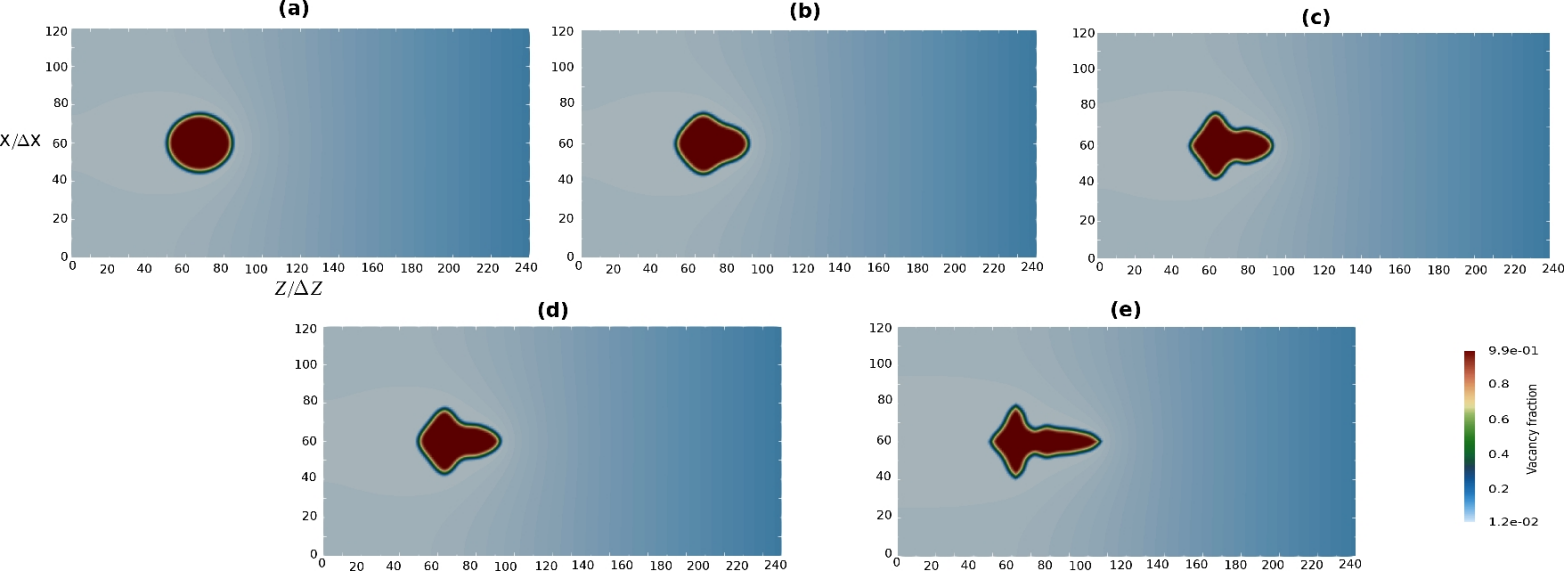
Impact of anisotropy strength of surface energy and interface mobility at $D_{\textrm{V}} =D_{\textrm{V}}^0/5$, $k=100$. (**a**) Isotropic surface energy and mobility, $\varepsilon _4=0$. (**b**) Isotropic mobility and anisotropic surface energy, $\varepsilon _4=0.023$. (**c**) Isotropic mobility and anisotropic surface energy, $\varepsilon _4=0.05$. (**d**) Anisotropic surface energy and mobility, $\varepsilon _4=0.023$. (**e**) Anisotropic surface energy and mobility, $\varepsilon _4=0.05$.

The results of the calculations without and with kinetic anisotropy for different surface anisotropy strengths are illustrated in Fig. 4. It is evident that increasing anisotropy strength of both surface energy and mobility will increase the instability and promote developing of dendritic arms.

(ii) Impact of supersaturation/diffusion coefficient of vacancies

To analyze the impact of kinetics on the interface instability, we first define the growth velocity as a function of the supersaturation. Note that the phase-field model utilizes the interface mobility calculated based on the equation for the “thin interface limit” (see Eq. 5) to satisfy the Gibbs-Thomson condition on the interface with zero kinetic coefficient. Mullins and Sekerka calculated the growth velocity of a growing sphere for diffusion-controlled growth as a function of the undercooling at infinity (see Ref.^7^ for more details). Analogous to undercooling ($\Delta$) in equation 3.19 in Ref.^7^, the superstaturation ($\Omega _{\textrm{V}}$) can replace and consequently the growth velocity of a pore in case of the vacancy diffusion can be written as

16 $$\begin{aligned} v_n = \frac{D_{\textrm{V}}}{R_0} \left( \Omega _\textrm{V}-\frac{2d_0}{R_0}\right) , \end{aligned}$$

where $R_0$ is the radius of growing sphere, which is the void radius in our case, and $\Omega _{\textrm{V}}$ is the dimensionless supersaturation of vacancies in the bulk phase far from the moving interface,

17 $$\begin{aligned} \Omega _{\textrm{V}}=\dfrac{y_{\textrm{V}} -y^\textrm{eq,b}_{\textrm{V}}}{y^\textrm{eq,p}_{\textrm{V}}-y^\textrm{eq,b}_{\textrm{V}}}. \end{aligned}$$

Since the capillary length $d_0$ is much smaller then the void radius, the second term in brackets in Eq. (16) can be omitted.

On the other hand, the vacancies sites conservation (similar to mass conservation law ) implies that the steady-state growth velocity in 2D case should be correlated using accumulation rate as

18 $$\begin{aligned} y_{\textrm{V}}^\textrm{eq, p} L_p v_n^\textrm{2D} = \iint \limits _{A} \dot{q}^\textrm{DC}_{\textrm{V}} \, dx \,dz \approx \dot{Q}_{\textrm{V}}L_x, \end{aligned}$$

where $L_p$ is the averaged pore size, *A* is an area around the pore that is free from the neighboring pores, and $L_x$ is the box size in the *x* direction. Here, we also assume that all vacancies in this area would annihilate to the pores (see analysis in Ref.^22^).

Hence, the supersaturation of vacancies, $\Omega _{\textrm{V}}$, can be estimated as the ratio between the accumulation rate and the diffusion coefficient of vacancies as

19 $$\begin{aligned} \Omega _{\textrm{V}} = \frac{\dot{Q}_{\textrm{V}} }{ D_{\textrm{V}}}\frac{R_0 \, L_x}{ L_p}. \end{aligned}$$

Using this equation, the theoretical values of supersaturation of vacancies are calculated for all tests and specified in caption of the figures. For these calculations, we choose $L_p = 2R_0 =10$ $\upmu$m. As the supersaturation of vacancies is correlated with the ratio $\dot{Q}_{\textrm{V}} / D_{\textrm{V}}$, it can be enhanced by either increasing the accumulation rate or decreasing the diffusion coefficient of vacancies. In the following tests, we change the diffusion coefficient and keep the source rate constant. Importantly, for each diffusion coefficient, the interface mobility should change appropriately to ensure the thin interface limit condition (see Eq. 5).

**Fig. 5:**
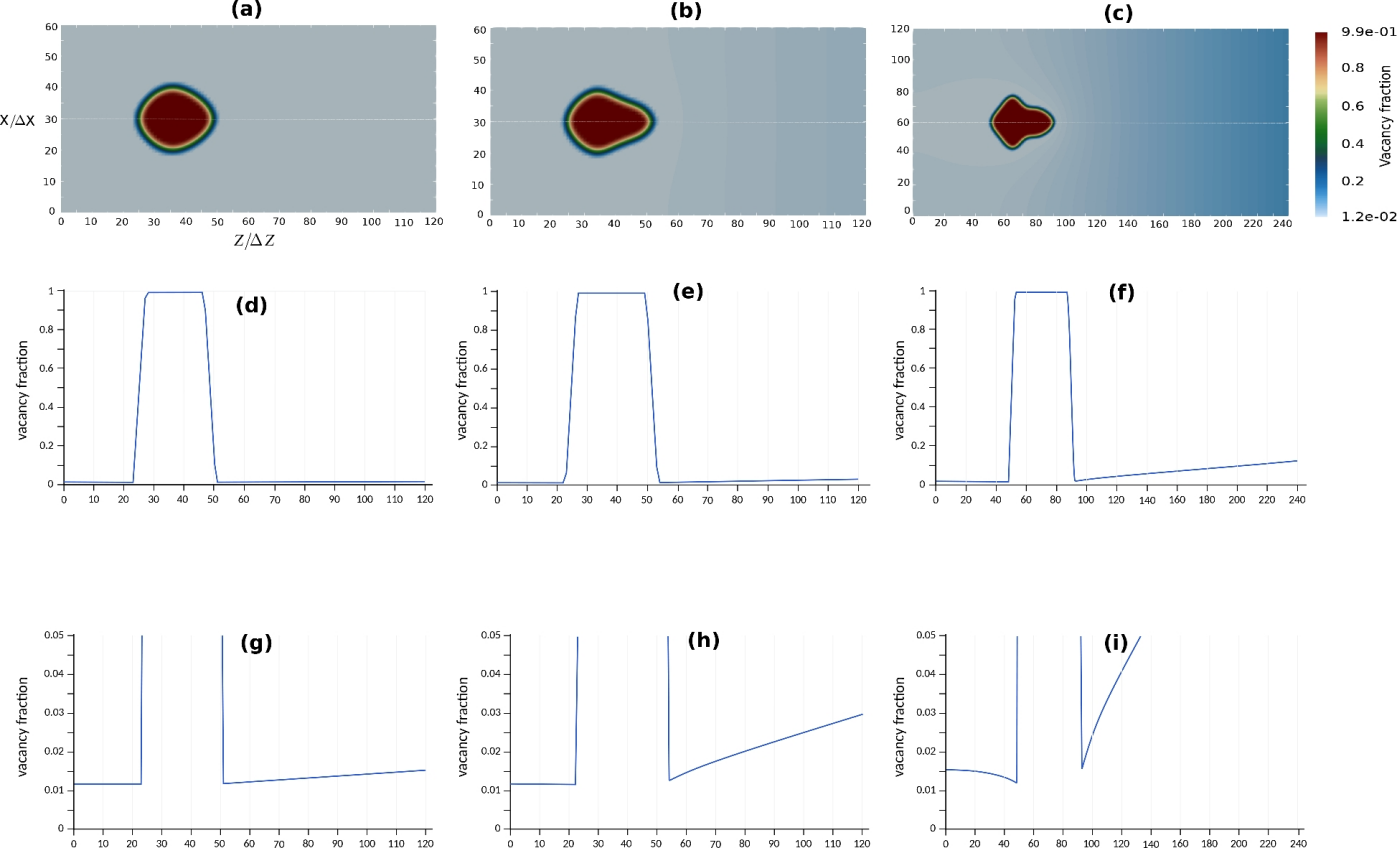
Impact of supersaturation/diffusion coefficient of vacancies at $k =100$. (**a**) $D_{\textrm{V}} =5D_{\textrm{V}}^0$, $\Omega _{\textrm{V}} =0.003$. (**b**) $D_{\textrm{V}} =D_{\textrm{V}}^0$, $\Omega _{\textrm{V}} =0.015$. (**c**) $D_{\textrm{V}} =D_{\textrm{V}}^0/5$, $\Omega _{\textrm{V}} =0.075$. (**a**–**b**) 2D view of vacancy site fraction. (**d**–**f**) Full views of vacancy fraction profile across the center line. (**g**–**i**) Close-up views of vacancy fraction profile across the center line.

The impact of the supersaturation of vacancies $\Omega _{\textrm{V}}$ on the morphological instability of voids is illustrated in Fig. 5. The morphology of the pores and the vacancy site fraction profile across the middle scanning line are shown. For each test, we calculate the supersaturation using Eq. (19). Concurrently, it is possible to calculate $\Omega _{\textrm{V}}$ directly using Eq. (21).

These results demonstrate that for different vacancy supersaturation, the voids exhibit symmetric dendritic-like forms that are elongated towards the vacancy flow, coming from the right boundary of the simulation box. The smaller diffusion coefficient for vacancies and higher supersaturation leads to arms formation tendency. As shown in Fig. 5c, where the diffusion coefficient is small ($D_{\textrm{V}}^0/5$) and the supersaturation is high ($\Omega _{\textrm{V}}=0.075$), a well-formed dendrite is obtained. The double resolution with the grid size $\Delta x_0/2$ at high supersaturation ensures the precision of the instability simulation. The void has smaller size owing to the smaller average growth velocity in comparison to cases (a) and (b).

(iii) Impact of partitioning

**Fig. 6:**
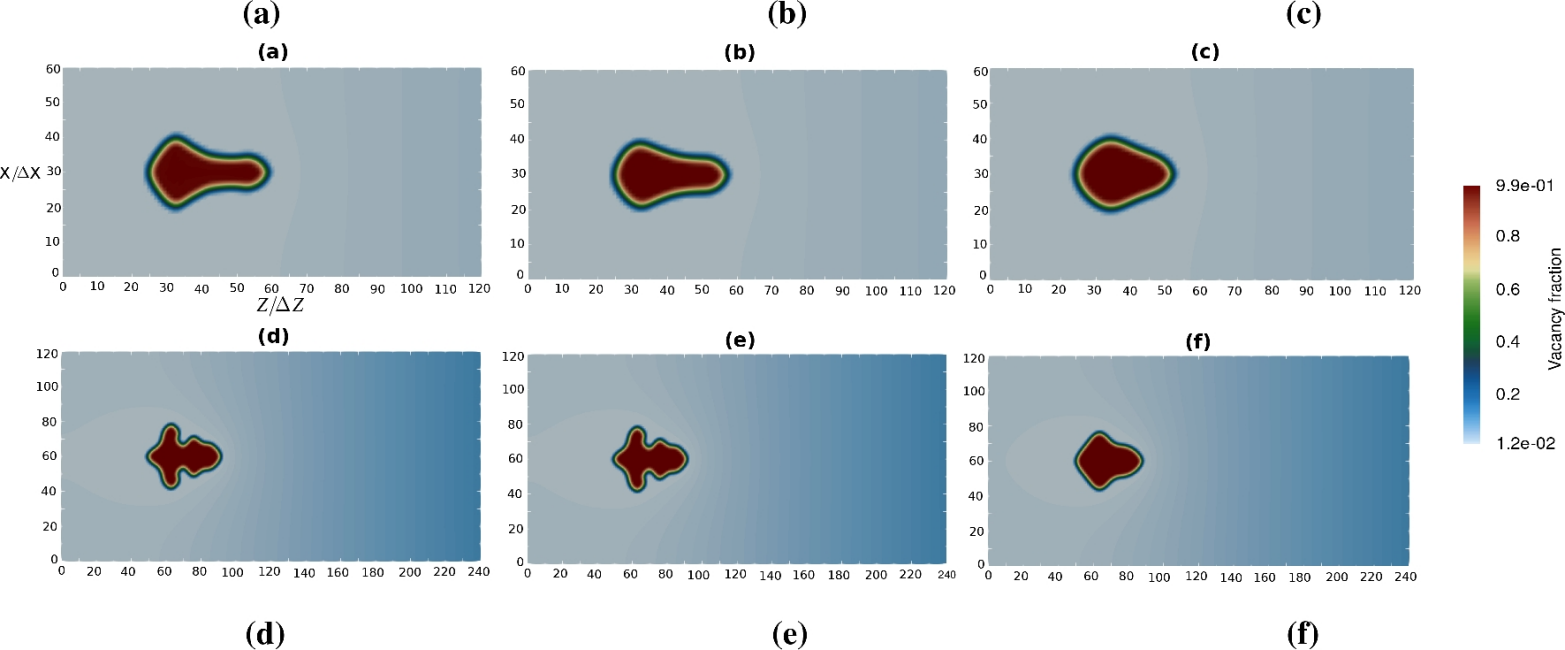
Impact of partition coefficient. (**a**–**c**) $D_\textrm{V} =D_{\textrm{V}}^0$, $\Omega _{\textrm{V}} =0.015$. (**d**–**f**) $D_\textrm{V} =D_{\textrm{V}}^0/5$, $\Omega _{\textrm{V}} =0.075$. (**a**,** d**) $k=1$. (**b**,** e**) $k=10$. (**c**,** f**) $k=100$.

The partition coefficient *k* is defined as the ratio between the thermodynamic factors in the pore phase (voids) and bulk phase $k = X_{\textrm{p}} /X_{\textrm{b}}$. It is responsible for the partitioning of the vacancies between the pore and bulk phases in the diffusion equation, and it also affects the driving force in the phase-field equation. The impact of the partition coefficient is shown in Fig. 6. Increasing the partition coefficient decreases the instability, i.e., it delays dendritic arm formation. For higher partition coefficients, the right tail of the dendritic shapes becomes less elongated (fat dendrites) in comparison to the cases with smaller partition coefficients.

(iv) Impact of source velocity

**Fig. 7:**
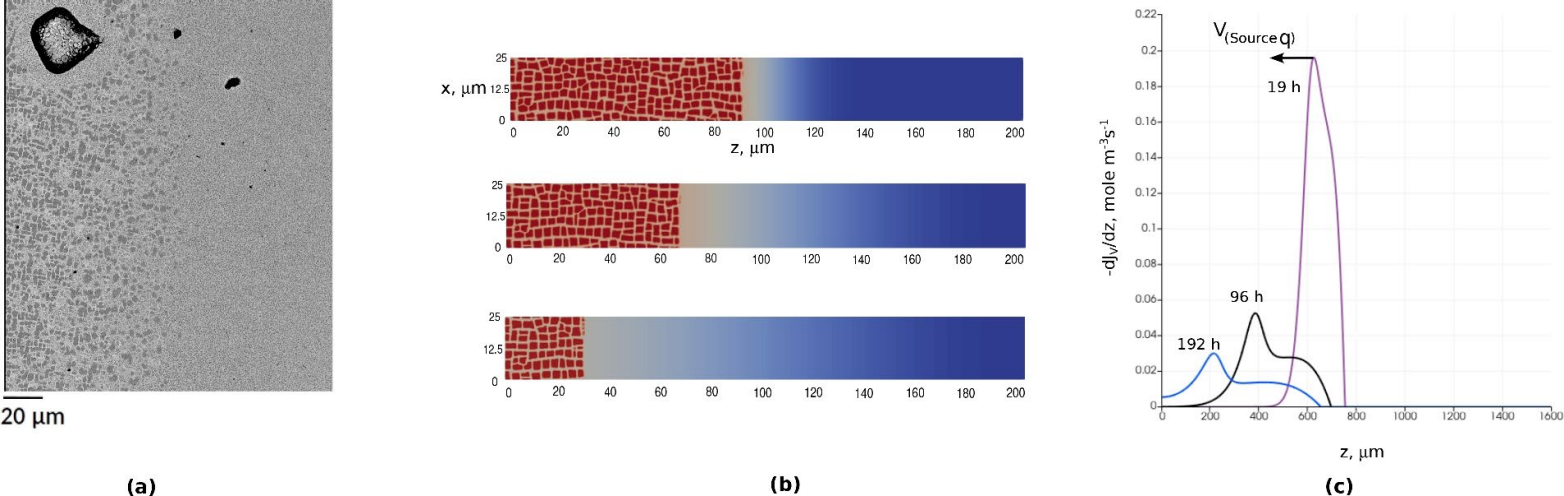
Velocity of vacancy source term. (**a**) SEM image of the region near left side of Matano plane, for diffusion couple CMSX-10/Ni annealed at T=1523 K for 192 h, representing a Kirkendall void (top left) at the front of the dissolution area of the $\gamma ^\prime$ phases (here is a horizontal diffusion couple compatible with diffusion couple vertically shown in fig. 2(a) ). (**b**) Simulations similar to Ref.^24^ show how the dissolution region of precipitates indicated by red color shift from right side to left side (upper simulation for 19 h, middle for 96 h, lower for 192 h). (**c**) The vacancy source term per volume, i.e., $\dot{q}^\textrm{DC}_{\textrm{V}}/ V_{\textrm{m}} = - \partial J_{\textrm{V}}/\partial z$, is plotted for several annealing times. The maximum of each curve indicates the $\gamma ^\prime$-dissolution front, moving at different velocities, $V_{q}$, during time.

In the preceding results, the vacancy source term $\dot{q}^\textrm{DC}_{\textrm{V}}$ was treated as immobile on the right side of the simulation box. However, simulations of elemental fluxes discussed in Refs.^21,22^ indicate that the vacancy source term has a strong maximum which appears on the boundary between the $\gamma /\gamma ^\prime$ and $\gamma ^\prime$-free regions. The EBSD image of the pores at the front of the dissolution of the $\gamma ^\prime$ phase in the CMSX-10 alloy can be found in Ref.^41^. During the heat treatment the $\gamma ^\prime$ precipitates dissolve and the boundary between the regions shifts. The time evolution of the vacancy source term and the shift of the boundary are illustrated in Fig. 7. To mimic this process in the next set of tests, we assume that the vacancy source is moving with a constant velocity, $V_q$, which is presumed to be proportional to the dissolution rate of $\gamma ^\prime$ precipitates, and we examine its impact on void morphology. The value of this velocity is evaluated using the experimentally defined growth rate constant of the $\gamma ^\prime$-free zone growth, which is smaller than roughly 7 $\times 10^{-10}$ m/s^21^.

**Fig. 8:**
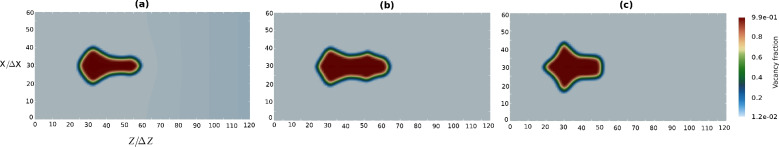
Impact of vacancy source term velocity at $D_{\textrm{V}} =D_{\textrm{V}}^0$, $\Omega _{\textrm{V}} =0.015$, $k =1$. (**a**) $V_q=0$. (**b**) $V_q=1\times 10^{-10}$ m/s. (**c**) $V_q=2\times 10^{-10}$ m/s.

The vacancy source velocity could significantly change the morphology instability trend. Figure 8 shows the shape of a single void at different source velocities, $V_q= \{0,1,2\}$ $\times 10^{-10}$ m/s. The movement of the vacancy source causes inward flux of vacancies from the top and bottom sides of the box, not just from the right side. This causes faster growth of dendritic arms in the *x*-direction, normal to the main direction of the diffusion couple.

(v) Impact of grid size

The choice of the discretization grid size, $\Delta x$, is crucial for modeling of interface instability and formation of the dendrite secondary-arms because it influences the ratio of stability length and interface width. To determine the appropriate resolution for each case, we perform simulations with different grid sizes, $\Delta x_0$ and $\Delta x_0/2$.

**Fig. 9:**
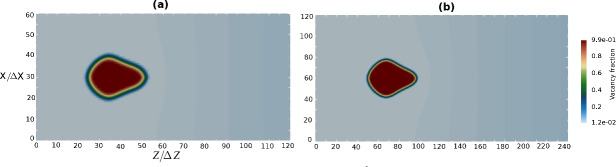
Impact of grid size at $D_{\textrm{V}} =D_{\textrm{V}}^0$, $k =100$, $\Omega _{\textrm{V}} =0.015$. (**a**) $\Delta x= \Delta x_0$. (**b**) $\Delta x=\Delta x_0/2$.

The results are illustrated in Fig. 9 for $D =D_{\textrm{V}}^0$, $k =100$. The barely detectable change in morphology is related to the slightly thicker width of the interface in case (a) compared with case (b); however, the growth velocity, affecting morphology instability, remains the same. Therefore, it is reasonable to choose grid size equal to $\Delta x_0$.

### Case II: 3D simulations

For the 3D simulations, we begin by simulating a single 3D void, aiming to achieve a dendrite shape. This 3D simulation parameters correspond to $D_{\textrm{V}}=D_{\textrm{V}}^0/5$, $M_{\phi }=M_{\phi }^{0}/5$, $\Delta x =\Delta y=\Delta z=\Delta x_0/2$, $\Delta t =\Delta t_0$, $\dot{Q}_{\textrm{V}}=2\times 10^{-11}\, {\textrm{m}}\,{\textrm{s}}^{-1}$. The box size is $80\times 50 \times 150\, \upmu {\textrm{m}}^3$. The initial pore nucleus is located at $x=40$ $\upmu$m, $y=25$ $\upmu$m, $z=45$ $\upmu$m. The diffusion coefficient and the accumulation rate are the same as in the test shown in Fig. 5c. In order to estimate the vacancy supersaturation in 3D, the relation for the conservation law in 2D, Eq. (18), should be modified to consider third direction as

20 $$\begin{aligned} y_{\textrm{V}}^\textrm{eq, p} L_p^2 v_n^\textrm{3D} = \iiint \limits _{V} \dot{q}^\textrm{DC}_{\textrm{V}} \, dx\, dy \,dz \approx \dot{Q}_{\textrm{V}}L_xL_y. \end{aligned}$$

where $v_n^\textrm{3D}$ is the growth velocity in 3D, *V* is the volume around the pore that is free from neighboring pores, and $L_x$ and $L_y$ are the box sizes in the *x* and *y* directions, respectively.

Using this relation, the vacancy supersaturation could be derived as

21 $$\begin{aligned} \Omega _{\textrm{V}} = \frac{\dot{Q}_{\textrm{V}} }{ D_{\textrm{V}}}\frac{ R_0L_xL_y}{ L_p^2}. \end{aligned}$$

Based on this formula and choosing $L_p=2R_0=16$ $\upmu$m, the vacancy supersaturation in 3D is estimated as $\Omega _{\textrm{V}} =0.08$. This value is obviously larger than the 2D case, therefore the 3D pore is expected to be larger than 2D pore.

**Fig. 10:**
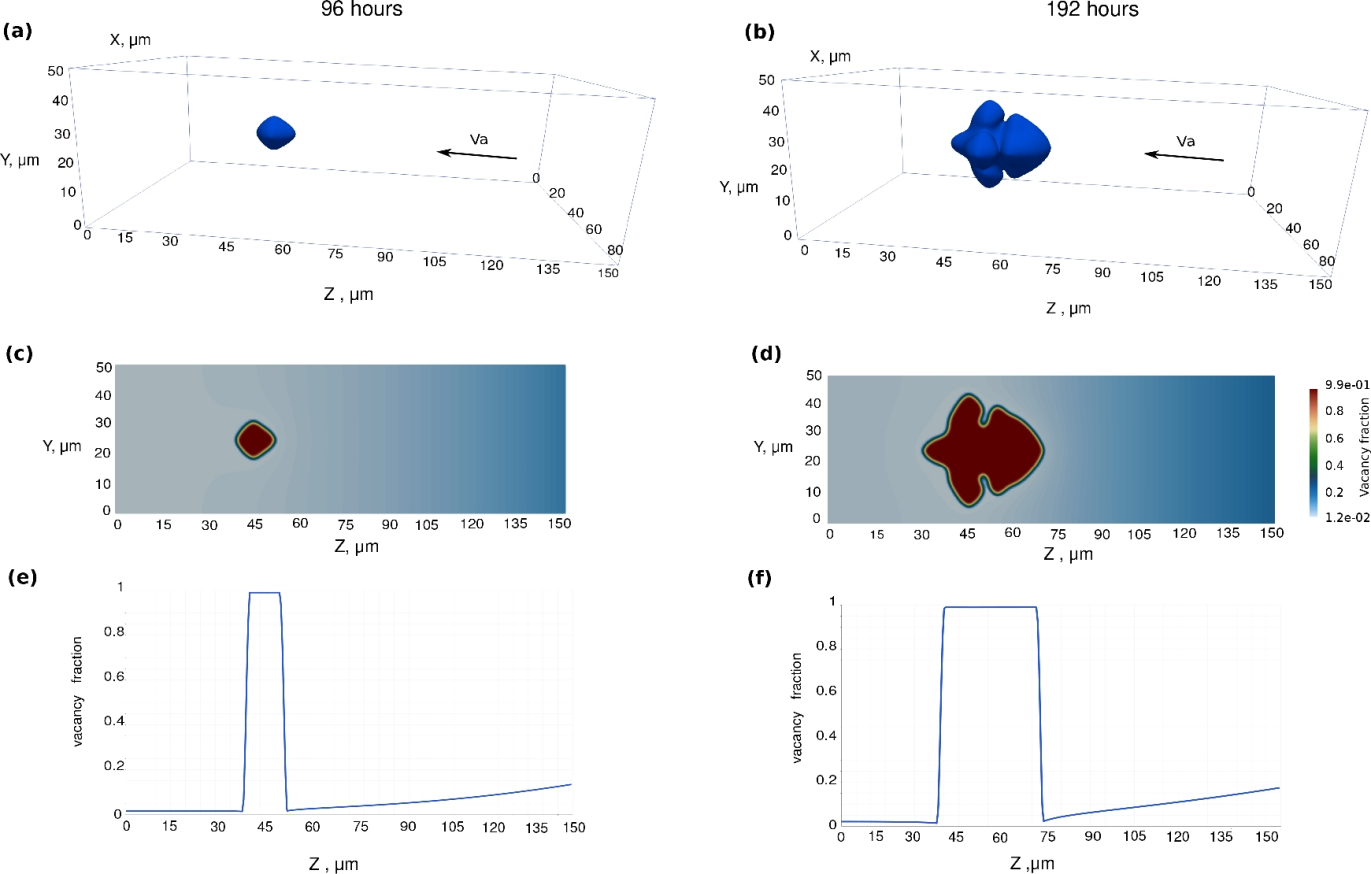
3D simulation results for a single void with parameters $D_{\textrm{V}} =D_{\textrm{V}}^0/5$, $k =100$ at intermediate and final times of annealing, i.e., 96 and 192 h. (**a**,** b**) 3D view of a single void. (**c**,** d**) Corresponding cross-sections representing the site fraction field of vacancies inside the void and in bulk alloy. (**e**,** f**) Vacancy site fraction profile over the scanning center line. The vacancy flux is from the right to left side.

Figure 10 displays the evolution of a single void for two different time steps, left ones (a,c,e) for 96 h and right ones (b,d,f) for 192 h. The cross-section of the 3D simulation on the *yz*-plane passing the middle of the *x*-axis and the corresponding vacancy fractions are plotted in this figures.

The vacancy flux from the right side of the box is pumped to the left side along the *z*-axis. The accumulation rate, $\dot{Q}_\textrm{V}$, is applied on the right side of the simulation box, similar to the 2D simulations. As shown, the semi-cubic void shape at the intermediate time (96 h) with an initial arm along the vacancy flux *z*-axis converts to a dendrite forming four additional arms in the *x* and *y* directions at the final time (192 h).

**Fig. 11:**
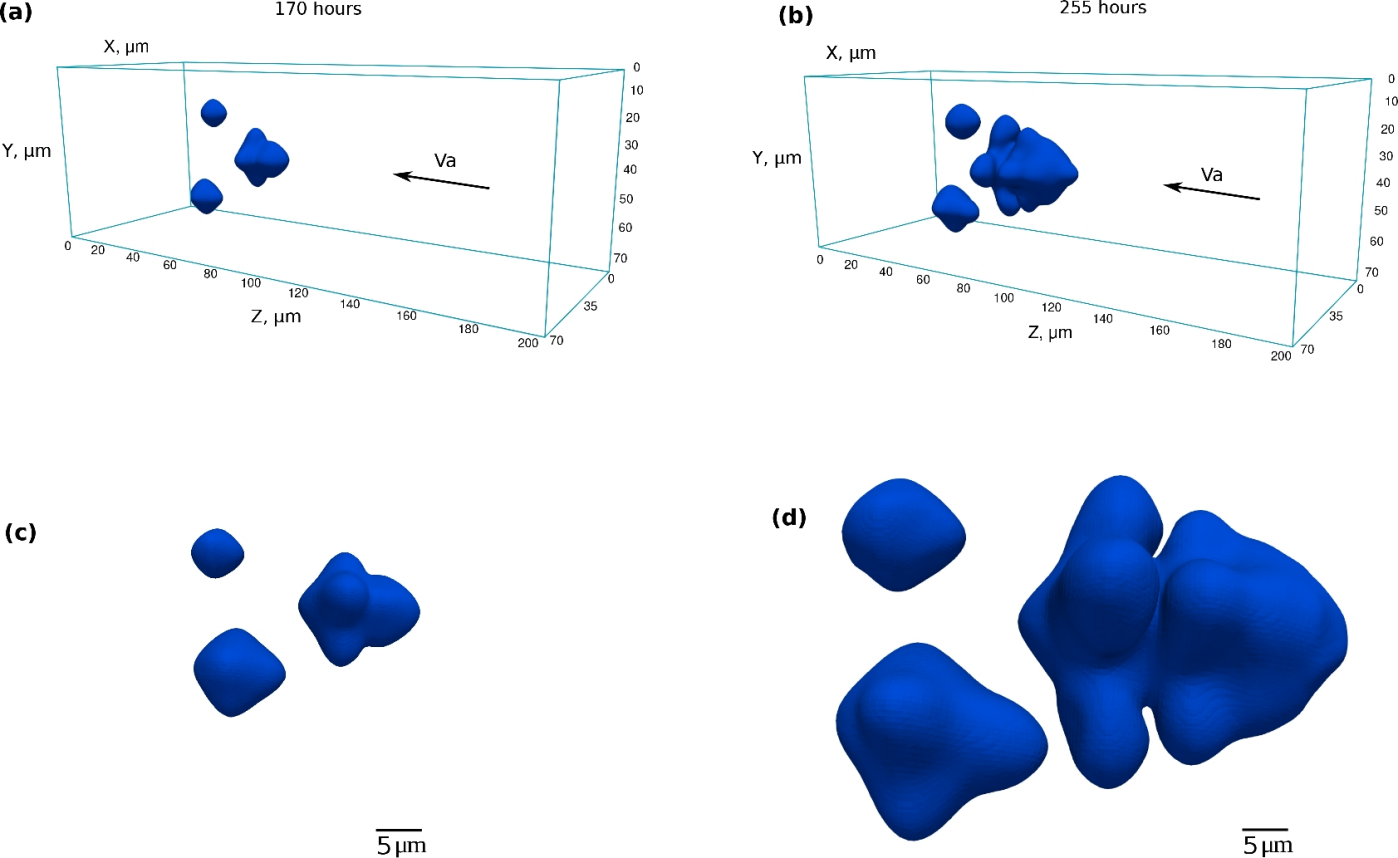
3D simulation of a three neighboring-void configuration for 170 h (left) and 255 h (right) of annealing. (**a**,** b**) 3D view of the simulation box. (**c**,** d**) Side view of voids pronouncing the asymmetrical morphology of two pores behind the front dendrite pore.

Figure 11 illustrates the case of three voids evolving next to each other on the left side of the diffusion couple after 170 h (left) and 255 h (right). The void sizes are very similar to the experimental ones and of the order of 20–40 $\upmu$m. As it can be observed in Fig. 11b,d, the large void on the front blocks the flow of vacancies and affects the shape of the voids behind it, making them smaller and asymmetrical relative to the sample axis and the axis of the dendrite.

### Case III: morphological instability in realistic-size sample

In this section, we describe simulations that closely approximate the real diffusion couple, comparable with Fig. 2a. These are used to reproduce the real distribution of void morphology changes, which depend on the distance from the Matano plane and their interactions with neighboring voids.

The 2D simulations of void growth in in the AlCoCrTaNi/Ni, hypothetically equivalent to CMSX-10/Ni,are performed by applying the model of void evolution in section *Methods*. Note that simulations in this part take into account the precise values of the vacancy source term, $\dot{q}^\textrm{DC}_{\textrm{V}}$, at each time step and space position, as defined by Eq. (10). This is in contrast to previous simulations, where approximations are used for the source term, such as a constant value and a stationary position. The model parameters are listed in Table 2. In the diffusion part, diffusion coefficients are calculated for each grid point and time step. The box size is 320 $\upmu$m $\times$ 1600 $\upmu$m, with the long *z*-axis in the direction of the diffusion couple.

The pore nucleation is modeled using a random method of nucleation with sites of varying activity (nucleation barrier) and incubation period depending on the structure of defects. For the nucleation barrier we choose different values for the source term, $\dot{q}^\textrm{DC}_{\textrm{V}}$, in the region from $1\times 10^{-10}$ to $4\times 10^{-10}\, {\textrm{s}}^{-1}$. Some pores develop at a later time due to a long incubation period.

**Fig. 12:**
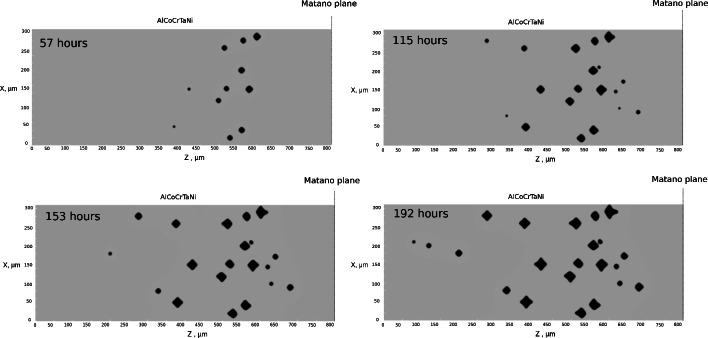
2D simulation of time evolution of void morphologies inside realistic-size diffusion couple AlCoCrTaNi/Ni represented by successive snapshots of 2D vacancy fraction field at different annealing times. The left half of the simulation box, where the Kirkendall pores appear is shown.

Figure 12 illustrates the time evolution of void morphologies. As time progresses, new pores continuously emerge at various distances from the Matano plane. The nucleation sites for these pores align with the position of the source term’s maximum, as depicted in Fig. 7.

**Fig. 13:**
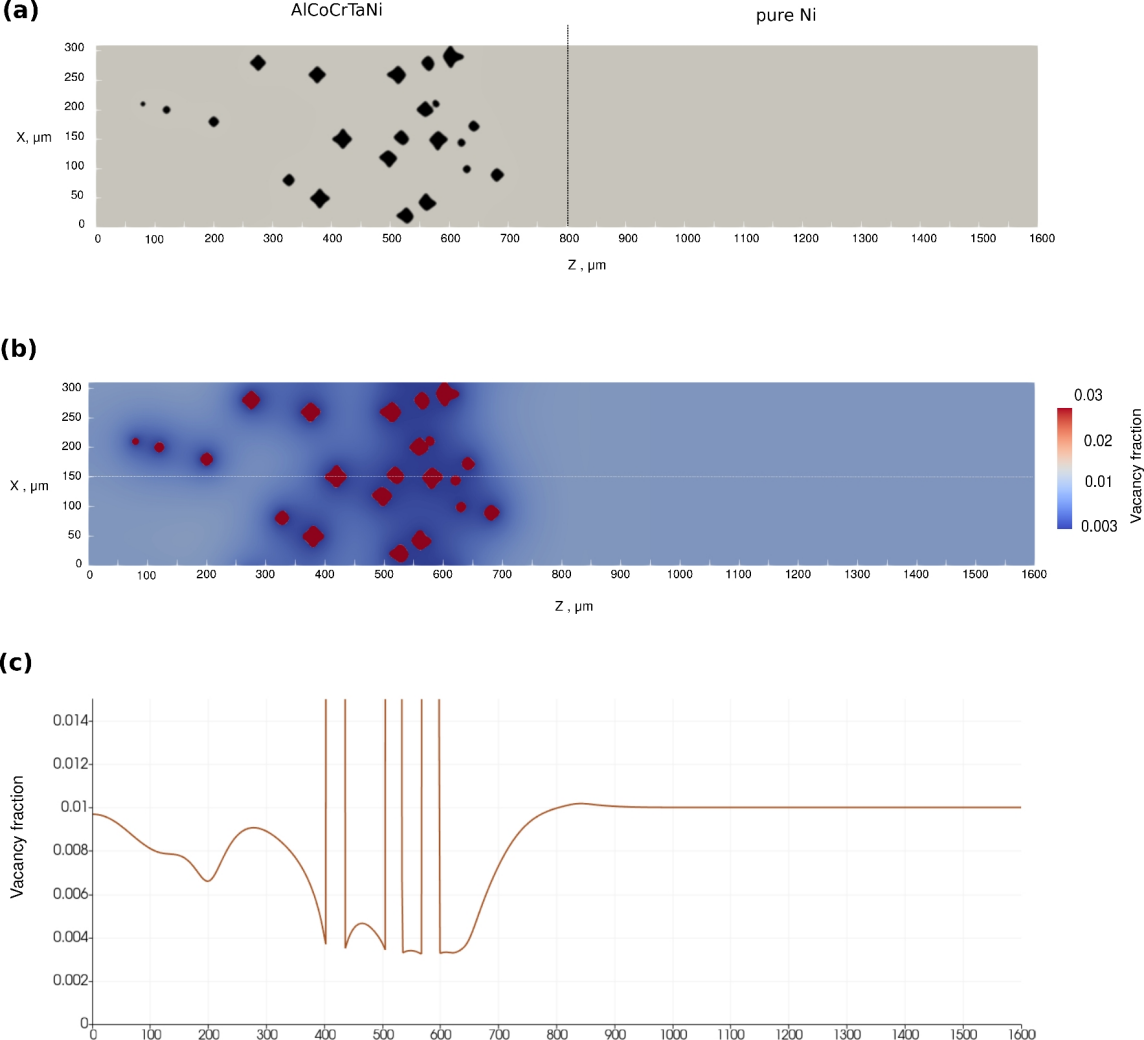
2D simulations of void morphologies inside realistic-size diffusion couple AlCoCrTaNi/Ni at last step. (**a**) 2D view of simulated neighbouring voids comparable with experimental observation for couple CMSX-10/Ni in Fig. 1c. (**b**) 2D vacancy site fraction field of vacancies, range (0.003-0.03) around the equilibrium vacancy fraction in matrix alloy (0.01). (**c**) 1D vacancy fraction profile across the central scanning line, representing fluctuations around pores, vacancy fraction range (0-0.014) around the equilibrium vacancy fraction in matrix alloy.

The results at 1523 K for 192 h are shown in Fig. 13. The 2D vacancy fraction distribution, which is zoomed near the equilibrium site fraction in bulk phase allows us to monitor the depletion of vacancy fraction near voids, which is especially intensive at the distances 200-300 $\upmu$m from the Matano plane. Interestingly, it is evident that fraction of voids is maximal at this region, corresponding to exactly the void distribution in the experiment as depicted in Fig. 2a. It can also be seen that the pore distribution and their density reproduce the experimental light micro-scope images of pores in Ref.^21^.

## Discussion

In this paper, we have investigated carefully how altering key parameters of the phase-field model could induce different morphologies.

Before we proceed with an in-depth discussion, several important points are worth emphasizing.

(i) The initial setup of the test in Case I begins with identifying the most critical parameter: anisotropy. The subsequent test simulations are conducted for different anisotropy strengths of surface energy with and without kinetic anisotropy. Taking into account the kinetic anisotropy is shown to make the dendritic form of voids more pronounced. For subsequent simulations, we set the value of the anisotropy strength for both surface energy and interface mobility as $\varepsilon _4=0.023$.
(ii) In all tests in Case I, the vacancy accumulation rate is assumed to be constant. That means that the amount of vacancies accumulated in the simulation box at a chosen time step is the same across all tests. Under these conditions, the varying diffusion coefficient of vacancies leads to variation of supersaturation (i.e., driving force), which affects consequently the the morphology instability of voids.
(iii) In Case III, the vacancy source term is calculated for each time step and each position relative to the Matano plane and depends on the current diffusion fluxes of components. That means that the amount of vacancies accumulated in the simulation box is a function of time and space. It causes vacancy supersaturation to vary along the diffusion couple, affecting the morphology instability of voids differently depending on the distance from the Matano plane.
(iv) In Case III, we use a fixed diffusion coefficient of vacancies, even though it can be a variable parameter. In fact, this value depends on the atomic mobilities of components, which change during the diffusion.

**Table 3:** Key parameters which affect the instability calculated at $\dot{Q}_{\textrm{V}}=2\times 10^{-11}$ m/s, $v_n^\textrm{2D} = 1.2\times 10^{-10}$ m/s, $v_n^\textrm{3D} = 5.5\times 10^{-10}$ m/s, $\operatorname {d}_0=4\times 10^{-10}$ m.

	Case I (2D)			Case II (3D)
$D_{\textrm{V}}$	$5D_{\textrm{V}}^0$	$D_{\textrm{V}}^0$	$D_{\textrm{V}}^0/5$	$D_{\textrm{V}}^0/5$
$\Delta x$	$\Delta x_0$	$\Delta x_0$	$\Delta x_0/2$	$\Delta x_0/2$
$l_{\textrm{dif}}$, $\upmu$m	3300	1000	130	30
$\lambda _s$, $\upmu$m	6.3	2.8	1.25	0.27
$\Omega _{\textrm{V}}$, Equation (17)	0.003	0.020	0.090	0.19
$\Omega _{\textrm{V}}$, Equations (19), (21)	0.003	0.015	0.075	0.08

Here, we explain the behavior of void morphology in Case I in more detail. According to Mullins and Sekerka’s linear stability analysis^7^ for a growing spherical precipitation, the stability length defines the minimum size of the sphere, whose interface is stable. If the size exceeds the stability length, the sphere becomes instable, so that in the case of cubic anisotropy it transforms to a dendritic shape with evolving arms. After this stage, stable dendritic growth can be observed if the diffusion length is equal to or greater than the tip radius, which in turn is determined by the vacancy supersaturation and anisotropy parameter.

In Table 3, the calculated key parameters, influencing the morphological instability, are listed. The average growth velocity for 2D and 3D cases is estimated by Eqs. (18) and (20), respectively; the diffusion length is calculated as $l_{\textrm{dif}}=\dfrac{2D_{\textrm{V}}^0}{v_n}$. The capillary length is defined as $d_0 = \dfrac{\sigma }{X_{\textrm{b}} (\Delta y_{\textrm{V}}^\textrm{eq})^2}$. Then we can estimate the stability length through Eq. (1) with $\alpha =\sqrt{0.5}$. In all cases considered in our simulations of single void evolution, the stability length is smaller than the average void size, resulting in interface instability and voids consequently transform to a dendritic shape. For the smaller diffusion coefficients, the stability length is smaller and the interface becomes instable at even a smaller void size. When the dendritic arms are formed, their tip radius is defined by the supersaturation and the anisotropy strength. In the case of dendritic tip grow, for smaller diffusion coefficients, the supersaturation is greater and thus, the tip radius is smaller, inducing sharper dendrites. If the supersaturation is smaller with a larger diffusion coefficient, then dendrites become fat.

The small diffusion coefficients for vacancies ($D_{\textrm{V}}^0/5$) leads to decreasing average growth velocity as it is shown in Fig. 5c. The vacancies accumulate near the right box boundary whereas there is no enough time for them to enter the pore phases, therefore the growth velocity decreases and supersaturation will increase. Consequently, $\Omega _{\textrm{V}}$ estimated by Eq. (17) (0.09) is larger than the one calculated by Eq. (19) (0.075). Both quantities, i.e., growth velocity and supersaturation, are not in their steady-state in this case.

As seen in results of Fig. 5 the higher supersaturation of vacancies amplifies morphological instability of voids, which is reflected by arms formation. This behavior can be explained by the fact that according to Eq. (19), the supersaturation is inversely proportional to the diffusion coefficient, whereas the stability length decreases with a decreasing diffusion coefficient as calculated via Eq. (1). The faster diffusion coefficient ensures an inward flux of vacancies from different sides of the box and not just from the side of the vacancy source, and hence the supersaturation decreases. Then, the stability length increases and eventually exceeds the void size, hindering arm formation. For a small supersaturation (0.003) at large diffusion coefficient ($5D_\textrm{V}^0$), the dendrite is fat and the tip radii of arms are large. This is because the stability length is nearly larger than the void size. For a larger vacancy supersaturation, the tip radii of the arms are smaller and the dendritic shape become sharper.

In general, the Mullins–Sekerka instability arises when small perturbations at the smooth interface between phases (e.g., the solid–liquid interface) begin to grow rather than decaying. In the context of a directed flux of vacancies in Case I, similar to solidification where there is transport of species, formation of a tiny bump on the interface can cause absorption of more vacancies because of the local concentration gradient. This further enhances the bump, leading to its growth. As a result, instead of being smooth and stable, the growing interface may become rough, and complex patterns such as dendrites will develop. The system becomes instable because the fluctuations (perturbations) at the interface are not smoothed out but are instead amplified by the directed flux. Here, the rate at which vacancies diffuse in the matrix alloy is a crucial parameter. If diffusion is slow compared with the growth velocity, the instability is more likely to occur, as shown in Figs. 5 and 6. In a similar way, the velocity at which the interface advances can either stabilize or destabilize the system. Faster growth velocities in comparison to diffusion can enhance instability, leading to more pronounced pattern.

In Case III, we observe significant changes in the void shapes along the diffusion couple, as shown in Figs. 12 and 13. Near the Matano plane, the pores have elongated arms in the positive z-direction, whereas those ones located at greater distance exhibit more symmetric forms or may even elongate in the opposite direction. Some of the pores resemble rounded pyramids, and certain pores look like octahedrals. Circular pores are visible near left end of diffusion couple probably because they begin forming at later stages, having less time to develop fully.

## Conclusions

In this work, phase-field modeling of the void growth, as developed in our previous work, combined with the CALPHAD-type data, is accompanied with Mullins-Sekerka instability theory for the study of morphological instability of Kirkendall voids in a Ni-based superalloy/Ni diffusion couple. The present 2D and 3D simulations demonstrate that the morphologies of voids are influenced by ratio between the vacancy diffusion coefficient and the vacancy source term, $\dot{q}_{\textrm{V}}^\textrm{DC}$, determining the growth velocity. A higher ratio results in lower supersaturation of vacancies, inducing more compact voids. Conversely, a lower ratio increases supersaturation and consequently cause the voids to form as sharp and branched dendritic structures. The directed flux of vacancies in the diffusion couple drives the elongation of one dendritic arm in the direction opposite to the vacancy diffusion, resulting in the formation of rounded pyramids, elongated droplets, and squid-like dendrites.

## Data Availability

The datasets used and/or analysed during the current study available from the corresponding author on reasonable request.
